# EphA4 expression promotes network activity and spine maturation in cortical neuronal cultures

**DOI:** 10.1186/1749-8104-6-21

**Published:** 2011-05-04

**Authors:** Meredith A Clifford, Jessleen K Kanwal, Rhonda Dzakpasu, Maria J Donoghue

**Affiliations:** 1 Department of Biology, Georgetown University, 37th and O St NW, Washington, DC 20057, USA; 2 The Interdisciplinary Program in Neuroscience, Georgetown University, 37th and O St NW, Washington, DC 20057, USA; 3 Department of Physics, Georgetown University, 37th and O St NW, Washington, DC 20057, USA; 4 Department of Pharmacology, Georgetown University, 37th and O St NW, Washington, DC 20057, USA; 5 Neurobiology Mentorship Program, Thomas Jefferson High School for Science and Technology, 6560 Braddock Road, Alexandria, Virginia, 22312, USA

## Abstract

**Background:**

Neurons form specific connections with targets via synapses and patterns of synaptic connectivity dictate neural function. During development, intrinsic neuronal specification and environmental factors guide both initial formation of synapses and strength of resulting connections. Once synapses form, non-evoked, spontaneous activity serves to modulate connections, strengthening some and eliminating others. Molecules that mediate intercellular communication are particularly important in synaptic refinement. Here, we characterize the influences of EphA4, a transmembrane signaling molecule, on neural connectivity.

**Results:**

Using multi-electrode array analysis on *in vitro *cultures, we confirmed that cortical neurons mature and generate spontaneous circuit activity as cells differentiate, with activity growing both stronger and more patterned over time. When EphA4 was over-expressed in a subset of neurons in these cultures, network activity was enhanced: bursts were longer and were composed of more spikes than in control-transfected cultures. To characterize the cellular basis of this effect, dendritic spines, the major excitatory input site on neurons, were examined on transfected neurons *in vitro*. Strikingly, while spine number and density were similar between conditions, cortical neurons with elevated levels of EphA4 had significantly more mature spines, fewer immature spines, and elevated colocalization with a mature synaptic marker.

**Conclusions:**

These results demonstrate that experimental elevation of EphA4 promotes network activity *in vitro*, supporting spine maturation, producing more functional synaptic pairings, and promoting more active circuitry.

## Background

Neural networks consist of cohorts of synaptically linked neurons whose well-coordinated function results in appropriate nervous system function. Continuous adjustment of neural networks in development and maturity promotes efficient and accurate cellular communication that is necessary for precise and rapid responses. An important means of establishing and tuning networks is spontaneous, non-evoked neural activity that occurs both as synaptic networks form and in maturity. Spontaneous periodic activity exists in many parts of the nervous system, including the retina [1], spinal cord [2], hippocampus [3], and neocortex [4,5]. In retinal development, spontaneous periodic activity is essential for retinotopic and eye-specific mapping [6,7], acting as a trophic influence [8], promoting axon pruning [9], and influencing responsivity to chemical cues [10]. A hypothesis regarding the role of spontaneous activity in neural organization and function is based on the importance of synaptic competition in defining functional circuitry and reflects the Hebbian logic that cells that fire together are more strongly connected; cells that are synaptically linked under non-evoked conditions communicate more effectively when stimulated [11-13]. Thus, elucidating that the molecular basis of effective cellular pairing during spontaneous firing is likely to highlight factors that establish and maintain neural networks throughout life. Our study focuses on molecular modulators of cortical neuronal function *in vitro*, specifically how a particular intercellular signaling molecule, EphA4, mediates cellular communication and impacts network activity.

### Cortical network dynamics

The emergence of spontaneous neuronal activity during development is essential for the establishment of neuronal connections, acting both to promote connectivity between particular cells and to facilitate future neuronal coordination [11,13]. Early activity in several neural systems, including the cortex, has a typical pattern of synchronized network bursts followed by periods of substantial electrical silence [12]. Interestingly, spontaneous activity present in neural structures *in vivo *emerges in cultures of neurons derived from these brain regions as they differentiate and form connections *in vitro *[14,15]. Synapses that act chemically via neurotransmitter signaling and electrically via cellular coupling both influence network synchronization [16]. For example, in cortical slices, gap junctions between neurons of the subplate and the cortical plate are critical for establishing early network oscillations, whereas in maturity, chemical signaling via glutamate receptors are the dominant mechanisms by which cortical networks synchronize [17]. Furthermore, while spontaneous activity can impact synaptic development, networks are plastic enough that there is adaptation when activity is experimentally blocked [12]. Spontaneous activity is generally independent of sensory activation; however, afferent inputs can act to synchronize network firing while local networks seem less sensitive to sensory input [15,17,18]. Still, intrinsic network dynamics can be modulated by different kinds of input (electrical manipulation via experimental introduction of low frequency stimulation shifted firing frequency, network burst shape, and temporal firing dynamics [19,20]), whereas genetic manipulation, modulation of a synaptic vesicle protein, synapsin, altered the overall excitability of the network without disrupting the fundamental pattern of bursting activity followed by silence [21,22]. These results support a model of spontaneous activity that is flexible and sensitive to both intrinsic shifts and environmental changes.

We employ the use of a multi-electrode array (MEA) recording system, consisting of an 8 × 8 grid of extracellular electrodes embedded within a silicon substrate, that is designed to record from *in vitro *neural networks at multiple locations such that the dynamics of network activity can be characterized over time and space [23]. For example, neural network activity at discrete physical sites can be characterized during a single recording session, thus revealing the firing properties of synaptically linked locations [18,24,25]. In addition, spatiotemporal patterns of network activity can be monitored from the same samples over several days, revealing emerging dynamics of neural firing [15,26]. The temporal resolution of the MEA approach allows the detection of multi-unit activity from large ensembles of neurons. Even in the absence of stimulation, spontaneous activity can be characterized using MEAs, both *in vivo *[27] and *in vitro *[28].

### Intercellular communication via Eph receptors and ephrin ligands

Eph proteins are transmembrane receptors that bind to ephrin ligands on neighboring cells [29,30]. Eph/ephrin engagement can trigger cellular responses in both cells, most often shifting the phosphorylation state of the receptor or ligand and then downstream targets, thus altering each protein's function and affecting subsequent signaling. For example, since Eph receptors are themselves tyrosine kinases, upon binding to a ligand, the receptors autophosphorylate and then phosphorylate downstream targets. This response within the receptor-containing cell is referred to as forward signaling [31]. In parallel, cellular changes occur in the ligand-containing cell - termed reverse signaling [32]. Although the ligand is not a kinase and thus cannot enzymatically promote signaling, partner enzymes facilitate signaling in ligand-expressing cells [33,34]. Initially, binding of Eph receptor to ephrins was considered to result in negative cellular outcomes, such as repulsion between interacting cells [35]. Recent, more nuanced studies, however, reveal that the outcomes of Eph/ephrin engagement exist on a continuum that includes attractive, neutral, and repulsive responses [36]. Additional factors, such as consideration of absolute and relative levels of signaling, identities and combinations of receptors and ligands, as well as the other intercellular signalers present, are likely to also be important in considering the outcomes of Eph/ephrin signaling [37,38].

In this study we focus on EphA4, an Eph receptor that is well-expressed within the developing forebrain [39,40] and localized to central synapses [41,42]. Previous studies revealed dichotomous roles for EphA4 in hippocampal synaptic form and function. In one set of studies, activation of EphA4 led to spine retraction *in vivo *[43,44], while another analysis that examined hippocampal neurons *in vitro *found that over-expression of the EphA4 kinase domain increased dendritic spine density while EphA4 knock-down reduced spine maturity [45]. Functionally, the absence of EphA4 limited hippocampal synaptic plasticity [46,47], while activation of EphA4 reduced hippocampal synaptic strength and implicated EphA4 in homeostatic plasticity [48]. Little is known, however, about the consequences of EphA4-mediated signaling on dendritic spines and network dynamics in the cerebral cortex.

We performed our studies in primary cultures of neocortical neurons derived from embryonic mice, and sought to characterize changes in spontaneous activity when EphA4 was over-expressed in a subset of the cells in culture. Our results demonstrate that elevated levels of EphA4 in some cultured neurons shifted spontaneous network dynamics, increasing both the duration of network bursts and the number of spikes per burst as well as altering the frequency of bursting in the cultures. In parallel with these physiological shifts, characteristics of dendritic spines of transfected neocortical neurons were also examined. Analyses revealed that neither the number nor the density of spines changed; however, dendritic spines on EphA4-overexpressing cortical neurons were significantly more mature than those on control cells. Together, enhanced network activity and more mature post-synaptic compartments support a role for EphA4 in promoting synaptic communication as developing cerebral cortical neurons form circuits.

## Results and discussion

### Expression of EphA4 in neocortical cultures alters spontaneous bursts

**Figure 1:**
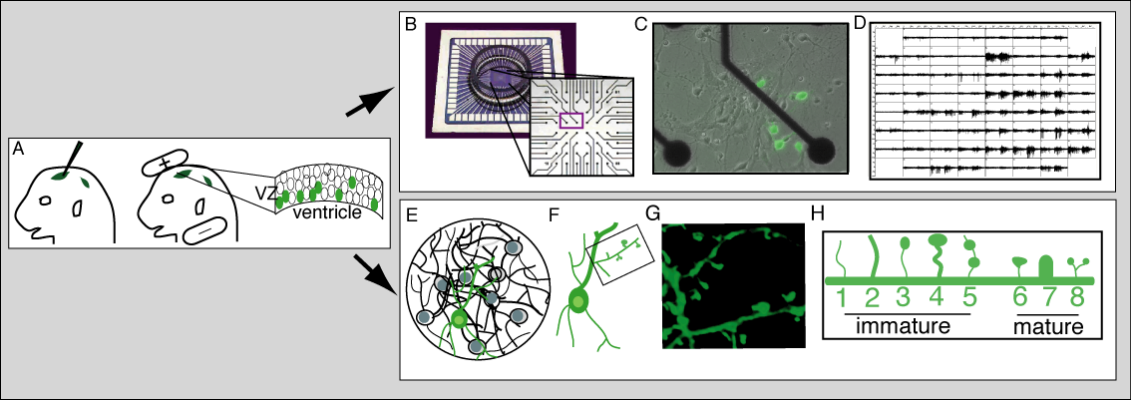
**Experimental design**. **(A) **To transfect cortical neurons, EUE was performed: a DNA solution (green) was injected into the ventricular system of an embryonic day 14.5 mouse brain (left), current was applied across the head (middle), and cells of the ventricular zone (VZ) transfected (right). The dorsal telencephalon was then dissected and cortical cells were dissociated and plated under conditions that promote neuronal differentiation, either into MEA chambers to monitor neural activity (top) or onto coverslips for morphological analyses (bottom). **(B) **MEA chambers contain an 8 × 8 grid of electrodes. **(C) **Transfected neurons plated into MEA chambers were visible within 24 hours of EUE. **(D) **Spontaneous activity arising from the developing cortical network was recorded from each electrode. **(E) **In culture, cortical cells differentiate and forge elaborate connections. **(F-H) **Dendritic spines from the second dendritic branch (F) of transfected neurons were imaged (G) and their shape was characterized according to the scheme shown (H), modified from Irwin *et al. *[55].

In order to begin to investigate the role of EphA4 in the emergence of cortical network activity, reliable transfection of cortical neurons was necessary. To accomplish this, embryonic cerebral cortex was transfected via *ex utero *electroporation (EUE) using DNA solutions that contained a plasmid encoding GFP with either an inert plasmid (control) or an expression vector encoding EphA4 (EphA4-transfected) that results in high levels of EphA4, visualized by ephrin binding, on transfected cells (Additional file 1). Following EUE, the dorsal telencephalon was dissected, cortical neurons were prepared and plated into MEA chambers and maintained under conditions that promote neuronal differentiation (Figure 1). Over time, the neurons matured and formed a complex neural network.

**Figure 2:**
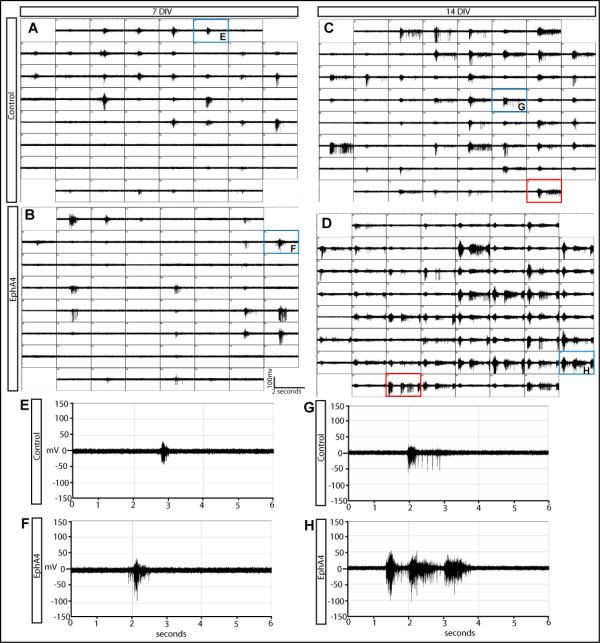
**Spontaneous activity of control- and EphA4-transfected networks**. **(A-D) **Screen shots of network bursts at 7 DIV (A,B) and 14 DIV (C,D) in control (A,C) and EphA4 (B,D) transfected networks. Each box represents spiking activity recorded from a single electrode over a period of 2 seconds. Boxes outlined in blue are magnified in (E-H) with the letter designations shown. The red boxes are examples of doublets and triplets in control- and EphA4-transfected cultures. **(E-H) **Single electrode recordings from control (E,G) and EphA4 (F,H) transfected networks at 7 DIV (E,F) and 14 DIV (G,H).

Spontaneous, rhythmic network activity emerged in these cortical neuronal cultures by 7 days *in vitro *(DIV), consistent with previous studies [49]. Recordings were analyzed from 7 DIV, when patterned activity was first evident, and from 14 DIV, when patterned activity was stable. Activity patterns persisted after 14 DIV, but neuronal health declined and these data were not analyzed. Mirroring other studies conducted on dissociated cortical cells, from 7 DIV onwards our cultures displayed intrinsic firing, characterized by periods of electrical silence punctuated with network bursting activity [5,15,50,51]. While the cellular basis of these silent periods remains to be elucidated, it is possible that after large network bursting, neurons become biochemically 'fatigued' and need time to recover before firing again [52]. As cultures matured, whether control- or EphA4-transfected, there were increases in the number of active electrodes, the burst duration, and the number of spikes per burst (Figure 2A-D). Despite being grossly similar, qualitative differences in network dynamics were immediately apparent between control- and EphA4-transfected cultures. In particular, EphA4-transfected cultures displayed noticeably longer bursts than those found in control networks (Figure 2E-H).

To quantify this difference, bursting activities from cultures at 7 and 14 DIV were analyzed. Analysis of bursts focused on three important and commonly assessed parameters: burst duration, number of spikes per burst, and interburst interval (IBI). Each parameter provides unique insights into the firing characteristics of the cells, which in turn reflect the maturity and regulation of the entire neural network; more mature cultures display increased neural activity [49]. Intraburst parameters (burst duration and spikes per burst) will be addressed first, followed by an examination of IBIs.

**Figure 3:**
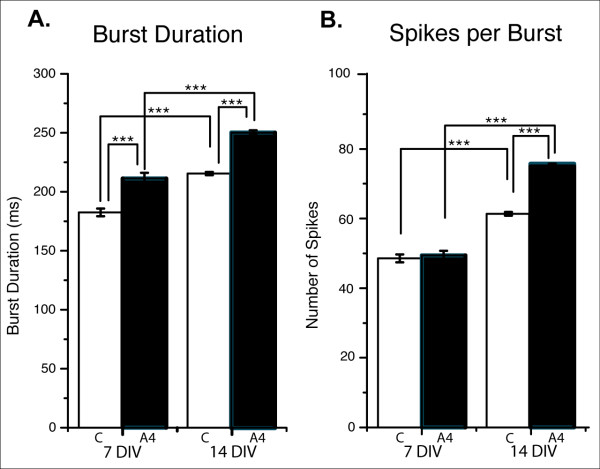
**Mean values for burst duration and spikes per burst at 7 and 14 DIV**. **(A) **Average burst duration in control (C, white) and EphA4-transfected (A4, black) primary cortical networks at 7 DIV (left) and 14 DIV (right). EphA4 transfected networks have significantly longer bursts than control networks at both time points. **(B) **Average number of spikes per burst in control (white) and EphA4-transfected (black) networks at 7 DIV (left) and 14 DIV (right). Bursts from EphA4-transfected networks contain significantly more spikes than control networks at 14 DIV, but not 7 DIV. Bursts at 14 DIV are significantly longer and have more spikes than bursts at 7 DIV in both groups. (***P < 0.0001 as determined using ANOVA, error bars represent the standard error of the mean).

Analysis of mean burst duration confirmed our qualitative impression: at 7 DIV, cells in cultures transfected with EphA4 fired longer bursts than those fired by cells in control cultures (mean = 211.2 ± 4.9 ms in EphA4 versus 182.5 ± 3.3 ms in control; Figure 3A). Similar numbers of spikes per burst were present in both conditions at 7 DIV (mean = 48.57 ± 1.2 spikes/burst for EphA4 and 47.69 ± 1.1 spikes/burst in control cultures; Figure 3B).

By 14 DIV, both control- and EphA4-transfected cultures demonstrated significantly increased spikes per burst and burst durations when compared to bursts exhibited by the same cultures at 7 DIV (Figure 3). EphA4-transfected cultures had both increased burst duration (mean = 248.9 ± 2.0 ms for EphA4 versus 214.2 ± 1.4 ms for control; Figure 3A) as well as number of spikes per burst compared to the control-transfected cultures (mean = 73.2 ± 0.8 spikes/burst in EphA4-transfected cultures versus 59.8 ± 0.5 spikes/burst in control-transfected cultures; Figure 3B). These increases are consistent with more mature, active networks when EphA4 levels are elevated.

**Figure 4:**
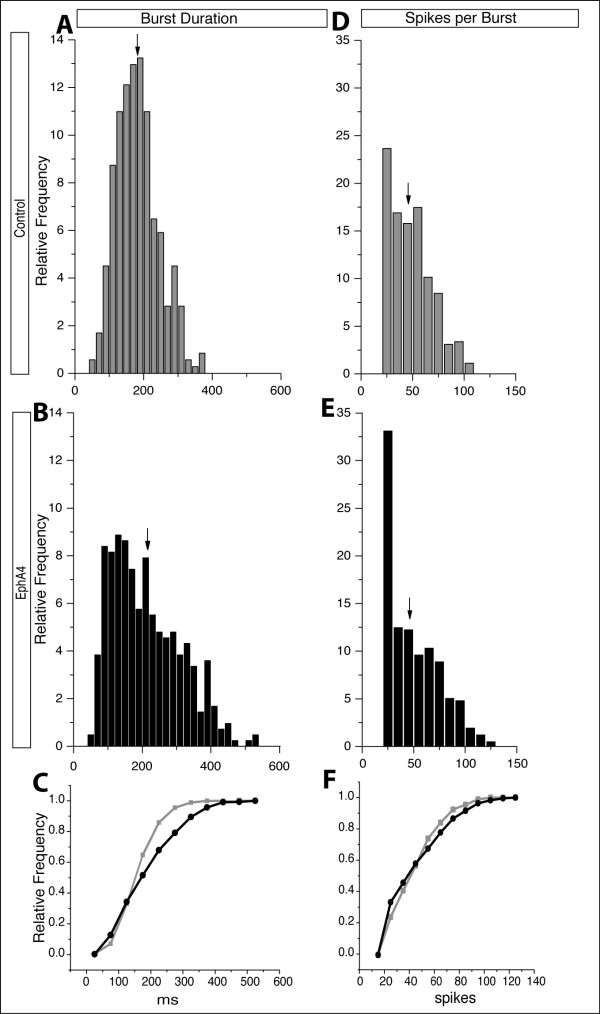
**Distribution histograms from 7 DIV cultures**. **(A,B) **Burst duration histograms from control (A) and EphA4-transfected cultures (B). EphA4-transfected cultures generate more long bursts compared to control networks. **(C) **Cumulative histogram of burst duration in control (grey) and EphA4 (black) depicts a shift towards longer burst durations in EphA4-transfected cultures. **(D,E) **Histograms of spikes per burst in control (D) and EphA4 (E) transfected networks demonstrate that despite similar mean values for spikes per burst, EphA4 networks have increased numbers of bursts with fewer spikes and bursts with more spikes than control networks, also demonstrated in the cumulative histogram in **(F)**. Arrows indicate means.

To further characterize bursting activity in control- and EphA4-transfected networks, distribution histograms of burst parameters were generated from data at both ages. At 7 DIV, the spread of burst durations in EphA4 networks was positively skewed, toward higher values, when compared to control-transfected cultures (compare Figure 4A with 4B,C). For example, approximately 65% of the bursts from control cultures were 200 ms or shorter, while a smaller proportion (less than 55%) of bursts from EphA4-transfected cultures fell into this category (Figure 4C).

Interestingly, there were also striking shifts in the distribution of values for spikes per burst in EphA4-transfected cultures versus control cultures. EphA4-transfected cultures produced bursts with either more or less spikes than bursts recorded from control cultures (compare Figure 4D and 4E,F). The combination of increases in both high and low values resulted in mean values that were not remarkably different between the two conditions (Figure 3B). The difference in the shape of the spikes-per-burst distributions could be an indicator of an irregular network when EphA4 is elevated in some neurons within a cortical circuit.

Effective network activity results from a fine balance between excitation and inhibition. Consistent with this concept, the alterations in network activity we observed when EphA4 signaling was elevated are likely to reflect unstable excitatory and inhibitory synaptic coordination [53,54]. Indeed, EphA4-transfected cultures had longer-lasting bursts and a more varied range of spikes per burst, causing elevated, albeit irregular, network activity. Since network regularity, a hallmark of well-functioning neural circuits, is perturbed when EphA4 is mis-expressed, our results support the idea that Eph signaling normally acts to maintain proper synaptic balance.

**Figure 5:**
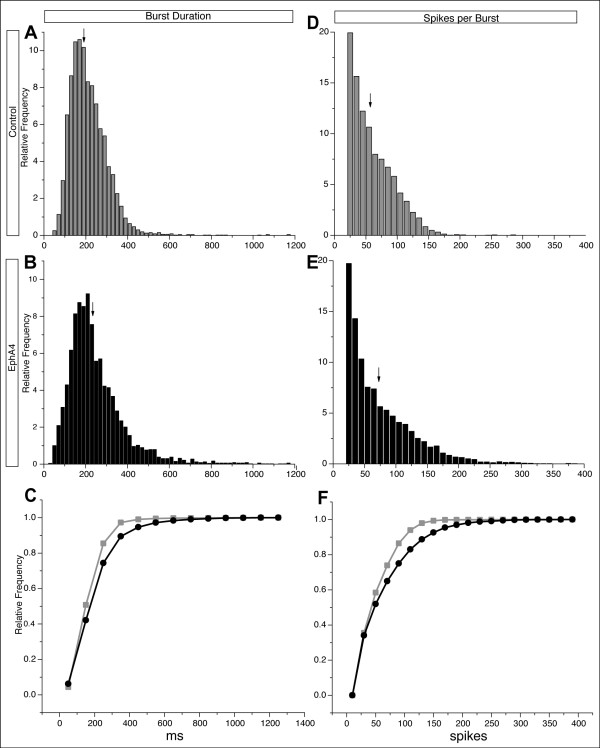
**Distribution histograms from 14 DIV cultures**. **(A,B) **Burst duration histograms from control (A) and EphA4 (B) transfected networks demonstrate increased numbers of bursts of longer duration in EphA4 networks. **(C) **The cumulative histogram of the burst durations in control (grey) and EphA4 (black) transfected cultures also demonstrates the shift towards higher values in the EphA4 cultures. **(D,E) **Histograms of spikes per burst in control (D) and EphA4 (E) transfected networks reveal that a higher proportion of bursts contain more spikes per burst in EphA4-transfected than in control networks. This shift is also apparent in the cumulative histogram in **(F)**. Arrows indicate means.

Analysis of data generated from cultures at 14 DIV yielded similar, yet more pronounced results. While control networks displayed normally distributed burst durations (Figure 5A,C), EphA4-transfected networks once again had a positively skewed distribution of burst durations in which a 'tail' consisting of higher values was present (Figure 5B,C), in keeping with an increase in burst duration average (Figure 3A and arrow in Figure 5A,B). Histograms depicting the distribution of spikes per burst were exponential for both networks (Figure 5D,E), but values for EphA4-transfected cultures were considerably larger (Figure 5F), again producing a 'tail' toward the right (Figure 5E), a shift towards higher values in the cumulative plot (Figure 5F), and a higher mean for EphA4- than control-transfected cultures (Figure 3B and arrow in 5E). Again, network changes when EphA4 levels were elevated were consistent with more spontaneous activity. Altogether, these results support a model in which a cortical circuit is more active when EphA4 is over-expressed.

By 14 DIV, both burst duration and the number of spikes per burst were higher in EphA4-transfected compared to control cultures. If the firing rates in EphA4-transfected cultures at 7 DIV were due to more inhibition, then the network appears to have compensated with increased excitation by 14 DIV, resulting in both elevated firing and longer bursts. Thus, our observations support gradual maturation of EphA4-transfected circuitry, eventually coordinating burst duration with firing rate under elevated conditions.

### Expression of EphA4 in neocortical cultures affects interburst parameters

**Figure 6:**
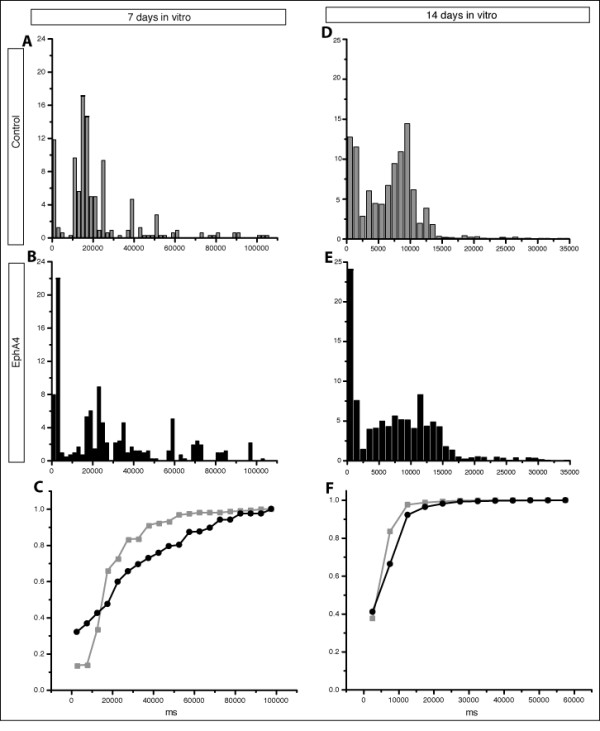
**Distribution histograms of interburst intervals at 7 and 14 DIV**. **(A-C) **IBI histogram for control (A) and EphA4 (B) transfected networks at 7 DIV. The distribution of IBIs is altered in EphA4 (black) compared to control (grey) transfected cultures as shown with the cumulative histogram (C). **(D-F) **IBIs from control (D) and EphA4 (E) transfected networks. Both networks show a bimodal distribution of intervals, with peaks at very short intervals and at longer intervals. The short interval peak is much higher while the long interval peak is flattened in EphA4- versus control-transfected cultures. The cumulative histogram (F) shows a slight shift towards increased IBIs in EphA4- transfected (black) cultures compared to control-transfected (grey) cultures.

Analysis of IBI at 7 DIV revealed that the regularity present in control cultures, characterized by two major peaks centered around 20 ms and around 20 s and a small number of IBIs corresponding to longer periods (Figure 6A), was altered when EphA4 levels were experimentally elevated (compare Figure 6A and 6B,C). In EphA4-transfected cultures, the first peak, centered near our designated maximum interspike interval of 20 ms, consists of the intervals between burst doublet and triplet motifs (see the recording outlined by the red box in Figure 2). Burst doublets and triplets consist of two to three successive bursts separated by intervals just over 20 ms in length, and are reflected by the high frequency of IBI values that comprise the first peak of the bimodal distribution. Each set of burst doublets and triplets is often separated by a long interval of silence, reflected by the second peak in the IBI distribution histogram, near 20 seconds. The second peak in IBIs of EphA4-transfected cultures was centered at a value slightly larger than the corresponding IBI peak in the control culture distribution. This may be due to the fact that the increase in network bursting required a longer refractory period before cells were again ready to start firing. Additionally, the peak centered near 20 seconds was more spread in these EphA4-transfected cultures, and more IBIs were found at higher values (Figure 6B).

Comparison of distribution histograms of IBIs at 14 DIV revealed other interesting differences between control- and EphA4-transfected networks (Figure 6D-F). Again, IBI values from both cultures are composed of two main peaks; however, at this time point one peak is centered near 20 ms and the second peak is centered close to 10 seconds. IBIs in control-transfected cultures exhibited fewer doublet and triplet bursts (based on the height of the first peak), but seem to be normally distributed around the second peak, while IBIs in EphA4-transfected cultures again deviated from the control with more frequent IBI values at the higher and lower ranges of the spectrum. This shift in IBIs is likely to reflect an increase in doublet and triplet bursts (boxed in red in Figure 2) along with longer bursts consisting of more spikes; more time may be required between bursts before the circuit is excitable again. The cumulative histogram in Figure 6F also demonstrates the slight shift towards longer IBIs when EphA4 levels are increased in a subset of the cultured cells.

Together, these results indicate that networks containing neurons with elevated levels of EphA4 are more active. While there are several possible explanations for this shift in network activity, EphA4's role in shaping dendritic spines, the main excitatory post-synaptic targets in the nervous system, in other neural systems led to the examination of spine characteristics in our transfected cortical neuronal cultures.

### EphA4 promotes dendritic spine maturity in neocortical neurons

**Figure 7:**
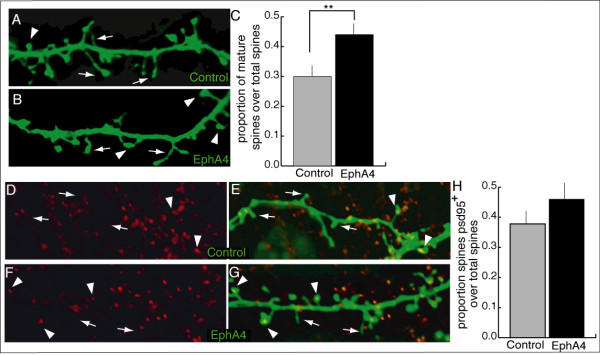
**Characteristics of dendritic spines in control- and EphA4-transfected neurons at 14 DIV**. **(A,B) **Dendritic shafts (horizontal) with protruding dendritic spines in control (A) and EphA4 (B) transfected neurons at 14 DIV. Arrowheads point to mature spines while arrows mark immature spines. **(C) **Neurons overexpressing EphA4 have a significantly higher proportion of mature dendritic spines compared to control neurons (**P < 0.01 as determined using ANOVA). **(D-G) **Dendritic spines of neurons transfected with actin-GFP and control (D,E) or EphA4 (F,G) and stained for PSD-95 (alone in (D,F)). Arrowheads point to spines with PSD-95 puncta while arrows mark spines without associated PSD-95. **(H) **A slight increase in the proportion of spines containing a PSD-95 puncta is seen in neurons overexpressing EphA4 compared to control neurons.

Cortical neuronal morphology was examined in order to discern differences between control- and EphA4-transfected neurons at 14 DIV, a time point at which spines are consistently present *in vitro*. Surprisingly, no differences in the morphology of neurons or the density of spines on either apical or basal dendrites were observed. Thus, the maturational state of spines on control- and EphA4-transfected neurons was examined. For this analysis, dendritic spines at preordained locations of apical dendrites of control- or EphA4-transfected neurons were visualized and spine morphology was characterized [55]. Specifically, each spine on the second branch of the apical dendrite (approximately 13 per branch) was classified as belonging to one of eight distinct spine shapes. Based on this dendritic spine morphology scale (Figure 1H), spine shapes one through five were considered immature while six through eight were considered mature [55]. The proportions of spines of a given shape and total mature and immature spines were then calculated for each cell and the data from cells of three experiments were summed. Altogether, this analysis demonstrated that neurons transfected with EphA4 had a significantly greater proportion of spines classified as 6 (mean control = 0.16 ± 0.02 versus EphA4 = 0.22 ± 0.02) and 7 (mean control = 0.08 ± 0.01 versus EphA4 = 0.12 ± 0.01), the most widely accepted mature spine shapes and the most common mature spine shapes we quantified, as well as total mature spines (sum of spines classified as 6 to 8) compared to control cells (mean control = 0.30 ± 0.03 versus EphA4 = 0.44 ± 0.03) (Figure 7A-C). Thus, neurons over-expressing EphA4 maintained more morphologically mature synapses than control-transfected neurons, perhaps explaining increased neuronal responsivity and consistent with more excitatory connections among neurons in the network. In keeping with increased spine maturation in EphA4 over-expressing neurons, there was also an increase in the proportion of spines with PSD-95 staining in EphA4-expressing neurons, an indicator of spine functionality (Figure 7D-H). The similarity in the extent of the increases in spine maturity and PSD-95 staining support a role for EphA4 in synaptic maturation.

In the developing cortex, EphA4 expression has been described and possible functions have been postulated [40-42,56], yet a role for this receptor in the development of neocortical neural networks has not been elucidated. Here, we report that elevating EphA4 in a subset of neurons in cerebral cortical cultures resulted in more active networks, evidenced by increases in both burst duration and the number of spikes per burst. How the small proportion of EphA4-transfected neurons in culture so profoundly shifted network dynamics in these studies was at first puzzling. Upon reflection, however, it became clear that this effect is consistent with the extent of interconnectedness in cortical systems, including our cultures. Indeed, single cells can have immense power in cortical systems [57-61]. For example, *in vivo*, the stimulation of a single cortical neuron can alter movement or behavior [57,60], while bursting of an individual cortical neuron is capable of initiating a transition into an entirely different brain state [61]. Differences between control- and EphA4-transfected cultures were consistent across the entire MEA and independent of the location of EphA4-transfected neurons, supporting a circuit-based model of EphA4's effect. The enhanced activity pattern in EphA4 over-expressing cultures was accompanied by increases in both the proportion of mature dendritic spines and the accumulation of the excitatory synaptic marker PSD-95 compared to control-transfected neurons. These findings are consistent with recent data linking a linear increase in synaptic puncta with an exponential increase in spike rates in dissociated neuronal cultures [62]. The parallels between the shifts observed in cortical neuronal network activity and dendritic spine characteristics when EphA4 is up-regulated with results from other neural systems [45,46] lead to the conclusion that Eph-mediated signaling may be an important means of modulating synaptic form and function across the nervous system.

## Conclusions

Our results demonstrate that elevated expression of EphA4 in a subset of cortical neurons in culture increased network activity, resulting in time-dependent increases in burst durations and spikes per burst and shifts in patterns of IBIs. Consistent with more active circuitry, morphological and immunohistochemical characteristics shifted: a larger proportion of dendritic spines were mature and aligned with PSD-95 in neurons with increased EphA4 signaling compared to control neurons. Together, these results implicate EphA4-mediated signaling in synaptic function and neural circuitry in cerebral cortical neurons.

## Methods

### *Ex utero *transfection, preparation, and culturing of neocortical neurons

Prior to plating, each MEA device, a 19 mm diameter chamber (Multichannel Systems, Ruetlingen, Germany), was cleaned and autoclaved. The MEA was first coated with 6.25 μg/ml poly-D-lysine for one night and then 6.25 μg/ml of laminin (Roche, Indianapolis, IN, USA) for the following night.

To transfect the cells, EUE of cortical neurons was performed. Briefly, following euthanasia of the mother, embryonic day 15.5 embryos were removed from the uterus and amniotic sac and a solution of DNA was injected into the ventricular system using a picospritzer (Figure 1). In all conditions, one-quarter of the injected DNA corresponded to a plasmid encoding GFP. For control cells, an inert plasmid (pSK^+^) was included, while for experimental cells a plasmid encoding full length EphA4 [40] was introduced. Current pulses delivered by tweezerodes (BTX, Holliston, MA USA) were then applied across the embryonic head in order to deliver DNA to the cells lining the ventricles. Following electroporation, the dorsal telencephalons were then dissected in ice cold HBSS (Gibco, Carlsbad, CA USA) supplemented with 0.5% D-glucose and 25 mM HEPES, the meninges were removed, and a single cell suspension was produced. Cortical neurons were plated onto MEAs at a density of 200,000 cells/MEA (705 cells/mm^2^) under conditions that promote differentiation (neurobasal media supplemented with 2% B27, 0.25% L-glutamine, 0.25% penicillin/streptomycin, 1 mM HEPES, and 5% horse serum). Using this method, 0.1 to 1% of cells in the resulting cultures were GFP^+^, visible 24 hours after transfection through a fluorescence microscope. Only cultures with an even distribution of cells after plating were used for this experiment. The cultures were covered with a gas permeable Teflon membrane to prevent evaporation of media and kept in a humidified chamber maintained at 37°C and 5% CO_2_. Cultures were monitored daily and provided fresh media every other day, or more frequently as the neurons matured. To feed cortical neurons without causing trauma associated with complete removal of overlying media, half of the media volume was removed and replaced with fresh media each time.

All animal use and care was in accordance with institutional guidelines, Georgetown's GUACUC protocols #06-022 and #09-020. CD-1 mice were from Charles River Laboratories (Germantown, MD USA).

### MEA recording system

Embedded in the center of each MEA chamber is an 8 × 8 square grid of 30 μm diameter titanium nitride electrodes with 200 μm inter-electrode spacing. This recording technique provides a non-invasive method of simultaneously recording electrical activity from up to 60 planar microelectrodes and allows for the monitoring of neural activity over several weeks.

Pilot experiments revealed that beginning at 7 DIV spontaneous neural activity was present in our cultures. Thus, 20 minutes of neural activity were recorded from each culture on day 7 and continued every 2 to 3 days for the following 2 weeks. To record activity, the MEA was placed in a 37°C preamplifier headstage that was connected to a computer (total gain was 1100X). The data were sampled at 25 kHz. Electrical activity was recorded and the data were used to quantify bursting dynamics generated from the spontaneous firing of neurons from each electrode (Figure 1). Cultures from three separate experiments were examined per group for each analysis. The shifts observed between control- and EphA4-transfected cultures were seen in each experiment and statistical analyses indicate consistency of results.

### Burst analysis

Cortical networks can display both single spiking and bursting activity. The bursting patterns, characterized by periods of continuous spiking at a relatively high rate with short interspike intervals, is most typical in mature dissociated cultures of cortical neurons. The duration of bursts can vary but generally last on the order of hundreds of milliseconds. In dissociated cortical cultures, bursts are separated by periods of little to no activity.

A burst detection algorithm was used to quantify the bursting patterns from the cortical cultures. After recording, the data were high-pass filtered at 25 Hz, and an Offline Sorter (Plexon, Inc., Dallas, TX, USA) was used to detect the time of each spike. Spike detection was based on a threshold, defined as 5 standard deviations away from the biological noise. This was individually performed for each channel since the noise level varied slightly from electrode to electrode. The time-stamps at which each spike occurred were saved and imported into a proprietary burst analysis program written in Matlab (Natick, MA, USA). For our experiments, a burst was defined to have a minimum number of 20 spikes and a maximum interspike interval of 20 milliseconds. Ten minutes of recording from 60 electrodes were analyzed for each time point. At least three experiments were analyzed in each condition.

### Spine analysis

To assess cell morphology, cortical neurons were transfected by EUE with the control plasmid or full-length EphA4 and an actin-GFP [63] plasmid to label spines. Cells were then plated on laminin-coated coverslips at the same density as the MEAs. After 14 DIV, the cells were fixed, immunohistochemistry was performed to enhance the GFP signal (rabbit anti-GFP, 1:3,000; Invitrogen (Carlsbad, CA USA) and to label PSD-95 puncta (mouse anti-PSD-95, clone K28/43, 1:1,000; UC Davis/NIH NeuroMab Facility). Appropriate secondary antibodies were used (anti-rabbit 488, anti-mouse 555; Invitrogen) and the coverslips were mounted onto slides (four to six coverslips per slide) using prolong (Invitrogen). Using fluorescent microscopy (100X), characteristics of dendritic spines were assessed in both control- and EphA4-transfected cortical neurons (n = 25 per experiment, 3 experiments). Mature and immature spines were categorized using a dendritic spine morphology rating scheme [55]. Each unique spine shape was classified as type 1 through 8, with types 1 to 5 considered immature and types 6 to 8 considered mature spines. Blinded to the EphA4-transfected condition, two investigators independently characterized the shape of spines located on the second branch of the apical dendrite of transfected neurons. A subset of the neurons was imaged and examined for the presence or absence of a PSD-95 puncta on each spine (n = 17 cells).

## Abbreviations

DIV: days *in vitro*; EUE: *ex utero *electroporation; GFP: green fluorescent protein; IBI: interburst interval; MEA: multi-electrode array.

## Competing interests

The authors declare that they have no competing interests.

## Authors' contributions

MAC and JKK participated in experimental design, conducted the experiments, analyzed the data, and wrote the manuscript. RD contributed the MEA system, her expertise on the MEA experimental design and data analysis, and provided editorial comments. MJD participated in experimental design, supervised the experiments, data interpretation, and wrote the manuscript. All authors read and approved the final manuscript.

## Supplementary Material

Additional file 1 **EphA4 transfected cells bind ephrin ligand**. **(A-H) **COS7 cells visualized with fluorescent Nissl stain (A,E) were transfected with GFP and a control construct (pSK^+^) (A-D) or EphA4 and GFP (E-H), fixed, then exposed to 3 μg/ml unclustered, recombinant ephrin-A5-Fc ligand for 1 hour at room temperature (as in Gale *et al. *[29]). Anti-human Fc antibody was used to detect the ligand that bound to endogenous or exogenous receptors (C,G). Cells overexpressing the full-length EphA4 were able to bind ligand much more readily than cells expressing the control construct as visualized in the merged images (D,H). Click here for file
