# Supplementary figures and images for: EphA4 expression promotes network activity and spine maturation in cortical neuronal cultures
Source: Neural Dev. 2011 May 4;6:21. doi: 10.1186/1749-8104-6-21 (PMC3100241; doi:10.1186/1749-8104-6-21)

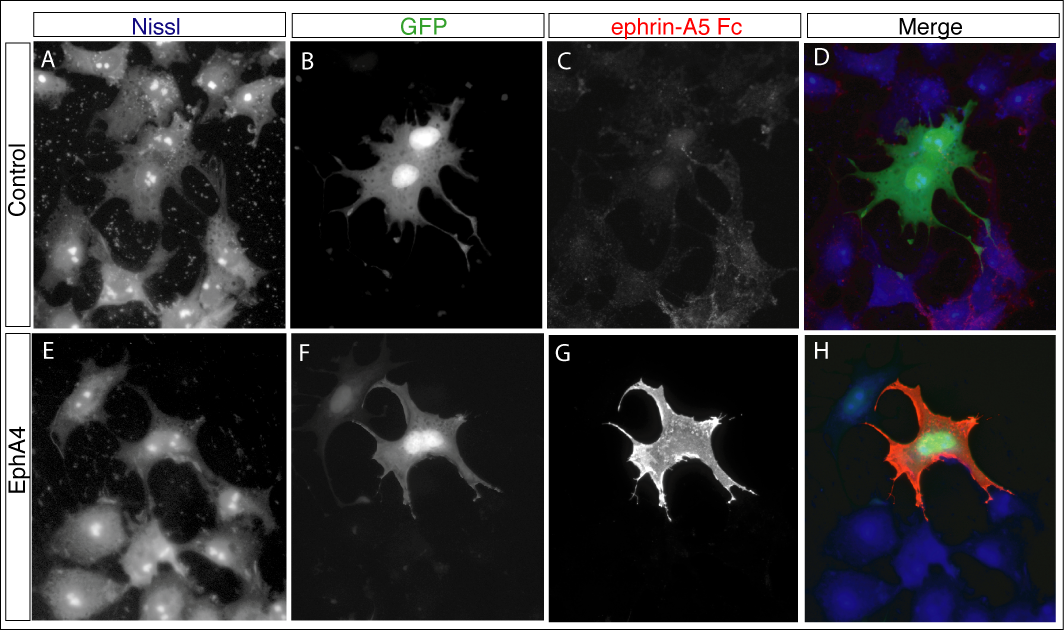

Supplement: Additional file 1 — EphA4 transfected cells bind ephrin ligand. (A-H) COS7 cells visualized with fluorescent Nissl stain (A,E) were transfected with GFP and a control construct (pSK+) (A-D) or EphA4 and GFP (E-H), fixed, then exposed to 3 μg/ml unclustered, recombinant ephrin-A5-Fc ligand for 1 hour at room temperature (as in Gale et al. [29]). Anti-human Fc antibody was used to detect the ligand that bound to endogenous or exogenous receptors (C,G). Cells overexpressing the full-length EphA4 were able to bind ligand much more readily than cells expressing the control construct as visualized in the merged images (D,H). [file 1749-8104-6-21-S1.PNG]
